# Increased sarcolemmal Na^+^/H^+^ exchange activity in hypertrophied myocytes from dogs with chronic atrioventricular block

**DOI:** 10.3389/fphys.2013.00322

**Published:** 2013-11-26

**Authors:** Marcel M. G. J. van Borren, Marc A. Vos, Marien J. C. Houtman, Gudrun Antoons, Jan H. Ravesloot

**Affiliations:** ^1^Heart Failure Research Center, Academic Medical Center, University of AmsterdamNetherlands; ^2^Department of Medical Physiology, Division Heart and Lungs, University Medical Center UtrechtNetherlands

**Keywords:** NHE-1, NBC, AE, CHE, compensated cardiac hypertrophy

## Abstract

Dogs with compensated biventricular hypertrophy due to chronic atrioventricular block (cAVB), are more susceptible to develop drug-induced Torsade-de-Pointes arrhythmias and sudden cardiac death. It has been suggested that the increased Na^+^ influx in hypertrophied cAVB ventricular myocytes contribute to these lethal arrhythmias. The increased Na^+^ influx was not mediated by Na^+^ channels, in fact the Na^+^ current proved reduced in cAVB myocytes. Here we tested the hypothesis that increased activity of the Na^+^/H^+^ exchanger type 1 (NHE-1), commonly observed in hypertrophic hearts, causes the elevated Na^+^ influx. Cardiac acid-base transport was studied with a pH-sensitive fluorescent dye in ventricular myocytes isolated from control and hypertrophied cAVB hearts; the H^+^ equivalent flux through NHE-1, Na^+^-HCO^−^_3_ cotransport (NBC), Cl^−^/OH^−^ exchange (CHE), and Cl^−^/HCO^−^_3_ exchange (AE) were determined and normalized per liter cell water and corrected for surface-to-volume ratio. In cAVB, sarcolemmal NHE-1 flux was increased by 65 ± 6.3% in the pH_*i*_ interval 6.3–7.2 and NBC, AE, and CHE fluxes remained unchanged. Accordingly, at steady-state intracellular pH the total sarcolemmal Na^+^ influx by NHE-1 + NBC increased from 8.5 ± 1.5 amol/μm^2^/min in normal myocytes to 15 ± 2.4 amol/μm^2^/min in hypertrophied cAVB myocytes. We conclude that compensated cardiac hypertrophy in cAVB dogs is accompanied with an increased sarcolemmal NHE-1 activity. This in conjunction with unchanged activity of the other acid-base transporters will raise the intracellular Na^+^ in hypertrophied cAVB myocytes.

## Introduction

Patients with left ventricular hypertrophy are at a higher risk to develop ventricular arrhythmias and sudden cardiac death (Bikkina et al., 1993; Oikarinen et al., 2001). This observation has been confirmed in various animal models with cardiac hypertrophy (Tomaselli and Marban, 1999), including dogs with chronic complete atrioventricular block (cAVB). The cAVB dog has compensated biventricular hypertrophy and a high susceptibility for drug-induced Torsade-de-Pointes arrhythmias (Vos et al., 1998) and sudden cardiac death (van Opstal et al., 2001). These potential lethal arrhythmias have been related to increased spatial (Verduyn et al., 1997) and temporal dispersion (Thomsen et al., 2004) of repolarization (Volders et al., 1999; Antoons et al., 2007).

An important factor that potentially contributes to action potential (AP) changes is the elevated intracellular Na^+^ (Na^+^_*i*_) in hypertrophied cAVB ventricular myocytes (Verdonck et al., 2003). High Na^+^_*i*_ is also reported in human hypertrophied myocardium (Gray et al., 2001). This condition reduces I_NCX_ inward current during the AP plateau phase thereby limiting AP duration lengthening. When Na^+^_*i*_ is high enough it may even promote NCX-mediated Ca^2+^ loading. Indeed, an increase Ca^2+^ influx through NCX was observed in cAVB (Sipido et al., 2000). The high Na^+^_*i*_ in hypertrophied cAVB myocytes has been attributed to an increased Na^+^ influx rather than impaired Na^+^ extrusion through the Na^+^/K^+^ pump(Verdonck et al., 2003). Recent studies indicate that the I_Na_ current (peak and late) is decreased and cannot underlie the increased Na^+^ influx in hypertrophied cAVB myocytes (Antoons et al., 2008).

A possible pathway for increased Na^+^ influx in hypertrophied myocardium is the Na^+^/H^+^ exchanger (NHE-1). The activity of NHE-1 is increased in a number of cardiovascular diseases and has been shown to be the major cause for the increased Na^+^_*i*_ concentration commonly observed in the hypertrophied failing hearts (Baartscheer, 2006; Bers et al., 2006). Inhibition of NHE-1 not only lowers Na^+^_*i*_ but also prevents cellular hypertrophy, ionic remodeling, delayed after depolarizations and ultimately the development of heart failure (Baartscheer et al., 2005). Although less-well studied and species dependent, the Na^+^-HCO^−^_3_ cotransporter (NBC) is another potential pathway for increased Na^+^ influx (van Borren et al., 2006; Yamamoto et al., 2007). In addition, the extent of Na^+^_*i*_ loading by NHE-1 and NBC largely depends on the supply of acid by the Cl^−^-dependent acid loaders such as, the Cl^−^/OH^−^ exchanger (CHE) and Cl^−^/HCO^−^_3_ exchanger (AE) (Chiappe de Cingolani et al., 2001; van Borren et al., 2006). However, as yet there is no literature on the identity and characteristics of the cardiac acid-base transporters in dog.

In this study we first confirm the presence of NHE-1, NBC, AE, and CHE in dog ventricular myocytes before we tested the hypothesis that increased sarcolemmal NHE-1 activity underlies the elevated Na^+^ influx in cAVB dogs with compensated biventricular hypertrophy. Here we report that dog ventricular myocytes exhibit NHE-1, NBC, AE, and CHE and that compensated cardiac hypertrophy in cAVB dogs is accompanied with an increased sarcolemmal NHE-1 activity. This together with unchanged sarcolemmal NBC, AE, and CHE activities will raise intracellular Na^+^ concentrations without significant consequences for the resting pH_*i*_.

## Materials and methods

Animal handling was performed in accordance with the “European Directive for the Protection of Vertebrate Animals used for Experimental and Scientific Purpose, European Community Directive 86/609/CEE” and under the regulations of “The Committee for Experiments on Animals” of the University of Utrecht, The Netherlands. All research was performed in accordance with the American PhysiologicalSociety's “Guiding Principles in the Care and Use of Animals.”

### Animal model and cell isolation

Total atrioventricular (AV) block (AVB) was created in adult dogs (Marshall, USA) of either sex (*N* = 6) by radiofrequency ablation of the AV node as previously described (Schoenmakers et al., 2003). At the time of sacrifice, AVB duration was 48 ± 7 days and body weight 24 ± 1 kg; normal control animals were weight matched (*N* = 5, 27 ± 2 kg). Normal and chronic AVB (cAVB) dogs received full anesthesia. After premedication (1 ml/5 kg: 10 mg oxycodone HCl, 1 mg acepromazine and 0.5 mg atropine, i.m.), sodiumpentobarbital (20 mg/kg i.v.) was given. Dogs were artificially ventilated with a mixture of oxygen, nitrous oxide (40:60%), and halothane (0.5–1% vapor concentration). Upon thoracotomy, heparin was administered i.v. The hearts were quickly excised and washed in cold cardioplegic solution. Heart weight/body weight was significantly larger in cAVB dogs (12.1 ± 0.4 g/kg, vs. 8.6 ± 0.3 g/kg in controls, *P* < 0.05). Single myocytes were enzymatically isolated from the midmyocardial layer of the left and right ventricular free wall, as previously described (Volders et al., 1999).

### Solutions

Tyrode's solution consisted of (mM) 140 NaCl, 5.4 KCl, 1.8 CaCl_2_, 1.0 MgCl_2_, and 5.5 glucose at pH 7.4 (37°C). The normal variant was buffered with 5.0 mM HEPES. In the CO_2_/HCO^−^_3_-buffered variant, 22.4 mM NaCl was replaced by NaHCO_3_ and the saline was gassed with 5% CO_2_ balanced with 95% air. For ammonium prepulses, 20 mM NaCl was replaced by NH_4_Cl. For acetate prepulses, 40 or 80 mM NaCl was replaced by NaAcetate (NaAc). In Na^+^-free Tyrode's solutions, Na^+^ was replaced by *N*-methyl-D-glucammonium (NMDG^+^) and Ca^2+^ was omitted to prevent Ca^2+^ loading via reverse mode NCX. In Cl^−^-free Tyrode's solutions Cl^−^ was replaced by gluconate. All salts were purchased from Merck (Darmstadt, Germany). The Na^+^/H^+^ exchanger inhibitor cariporide was kindly provided by Dr. Pünter (Aventis, Germany). Cariporide was prepared as 1000× stock in water.

### Intracellular H^+^ measurements

Myocytes were loaded with the fluorescent pH indicator carboxy-seminaphthorhodafluor-1 (SNARF, Molecular Probes) by exposing them for 10 min to 10 μM of the acetoxy methyl ester at 35°C. The inverted microscope was equipped with an apparatus for epi-illumination. Dye-loaded myocytes were excited with light of wavelength of 515 nm for 50 ms once every 3 s (75 W Xenon arc lamp). Intensities of the emitted light at wavelengths of 580 (I_580_) and 640 nm (I_640_) were recorded by two photomultiplier tubes. A rectangular adjustable slit ensured negligible background fluorescence levels. The I_580_/I_640_ ratio was calibrated by a series of precisely set pH solutions that contained 140 mM K^+^ instead of Na^+^ and 10 μM nigericin (Sigma) (van Borren et al., 2004).

### Computation of cytoplasmic H^+^ equivalent flux (J^+^_H_) per liter cell water

Intrinsic buffering power (β_int_) was determined in left and right ventricular myocytes from normal and cAVB dogs. We used the stepwise reduction in NH_3_/NH^+^_4_ technique as previously described by van Borren et al. (2004). In short, myocytes were exposed to series of nominally Na^+^ and Ca^2+^ free (replaced with N-methyl-D-glucammonium ions), HEPES buffered (pH 7.4) Tyrode's solutions containing decreasing amounts of NH_3_/NH^+^_4_. To minimize NH^+^_4_ entry via K^+^ channels, 2.0 mM BaCl_2_ was added, whereas addition of 1.0 mM CdCl_3_ prevented Ba^2+^ influx through L-type Ca^2+^ channels. β_int_ was calculated as –Δ[acid]_*i*_/ΔpH_*i*_ and assigned to the mean of the two pH_*i*_ values used for its calculation. The CO_2_/HCO^−^_3_ buffering power, β_CO_2__, was computed according to: β_CO_2__ = 2.3 × [HCO^−^_3_]_*o*_ × 10^(pH*i* − pH*o*)^, with [HCO^−^_3_]_*o*_ representing the extracellular HCO^−^_3_ concentration. The total buffering power, β_tot_, is defined as the sum of β_int_ and β_CO_2__.

To study the H^+^ equivalent flux (*J*^+^_H_) of the acid-base transporters, we imposed an acute acid load followed by an acute alkaline load on the myocytes and allowed them to recover from both. We fitted exponential functions to the recovering pH_*i*_ traces. From these functions we computed the time derivatives, dpH_*i*_/dt's, and multiplied these with the appropriate β_int_ or β_tot_ to obtain *J*^+^_H_. The first 4 min of the pH_*i*_ recovery from an alkaline load under CO_2_/HCO^−^_3_-buffered conditions were ignored to exclude out-of-equilibrium effects of the cytoplasmic buffering systems.

### Computation of sarcolemmal H^+^ equivalent flux (J^+^_H_) per unit membrane area

The sarcolemmal *J*^+^_H_ per unit area (amol/μm^2^/min) was computed by dividing the cytoplasmic *J*^+^_H_ by the surface to volume ratios. Atto (symbol a) is an SI prefix representing 10^−18^. Cell surface was estimated from the membrane capacitance C_m_ using a specific membrane capacitance of 10 fF/μm^2^. C_m_ was recorded with the whole-cell ruptured patch-clamp technique, the Axopatch 200B patch-clamp amplifier (Molecular Devices Corporation, Sunnyvale, CA, USA) and borosilicate glass patch pipettes (2–5 MΩ) filled with pipette solution containing (mM): 130 KCl, 10 NaCl, 0.5 MgCl_2_, 5 MgATP, 10 HEPES; pH 7.2 (5.5 KOH). C_m_ of right ventricular cAVB myocytes were significantly increased from 182 ± 10 pF (*n* = 29) to 216 ± 13 pF (*n* = 33) (*P* < 0.05), whereas the increase in C_m_ of left ventricular cAVB myocytes did not reach statistical significance, from 174 ± 6 pF (*n* = 44) to 187 ± 5 pF (*n* = 93), respectively. Length of left ventricular cAVB myocytes increased from 193 ± 2 μm (*n* = 197) to 225 ± 4 μm (*n* = 154) (*P* < 0.05) and of right ventricular cAVB myocytes from 200 ± 3 μm (*n* = 183) to 244 ± 6 μm (*n* = 154) (*P* < 0.05). Width of left ventricular cAVB myocytes increased from 27 ± 0.4 μm (*n* = 197) to 33 ± 1 μm (*n* = 154) (*P* < 0.05) and of right ventricular cAVB myocytes from 26 ± 0.4 μm (*n* = 183) to 35 ± 1 μm (*n* = 154) (*P* < 0.05). Cell volume was estimated from the morphologic data, assuming cylindrical cell shapes. The surface-to-volume ratios of left ventricular cAVB myocytes was reduced by 38%, from 0.16 ± 0.01 to 0.10 ± 0.01 μm^−1^, and from 0.17 ± 0.02 to 0.09 ± 0.01 μm^−1^, a decrease of 47%, on the right side. By dividing the cytoplasmic *J*^+^_H_ (mM/min) by surface-to-volume ratios we arrived at the sarcolemmal *J*^+^_H_ per unit area of cell membrane per minute (amol/μm^2^/min).

### Statistics

Results are expressed as mean ± standard error (SE). We conducted statistical analyses (linear regression model with repeated measurements, ANOVA Student's *t*-tests) using SPSS® software. Two sets of data were considered significantly different if the *P*-value of these tests was <0.05. The capital “N” represents the number of hearts used. Lower case “n” represents the number of cells measured.

## Results

### Identification of sarcolemmal acid-base transporters in normal dog ventricular myocytes

**Figure 1:**
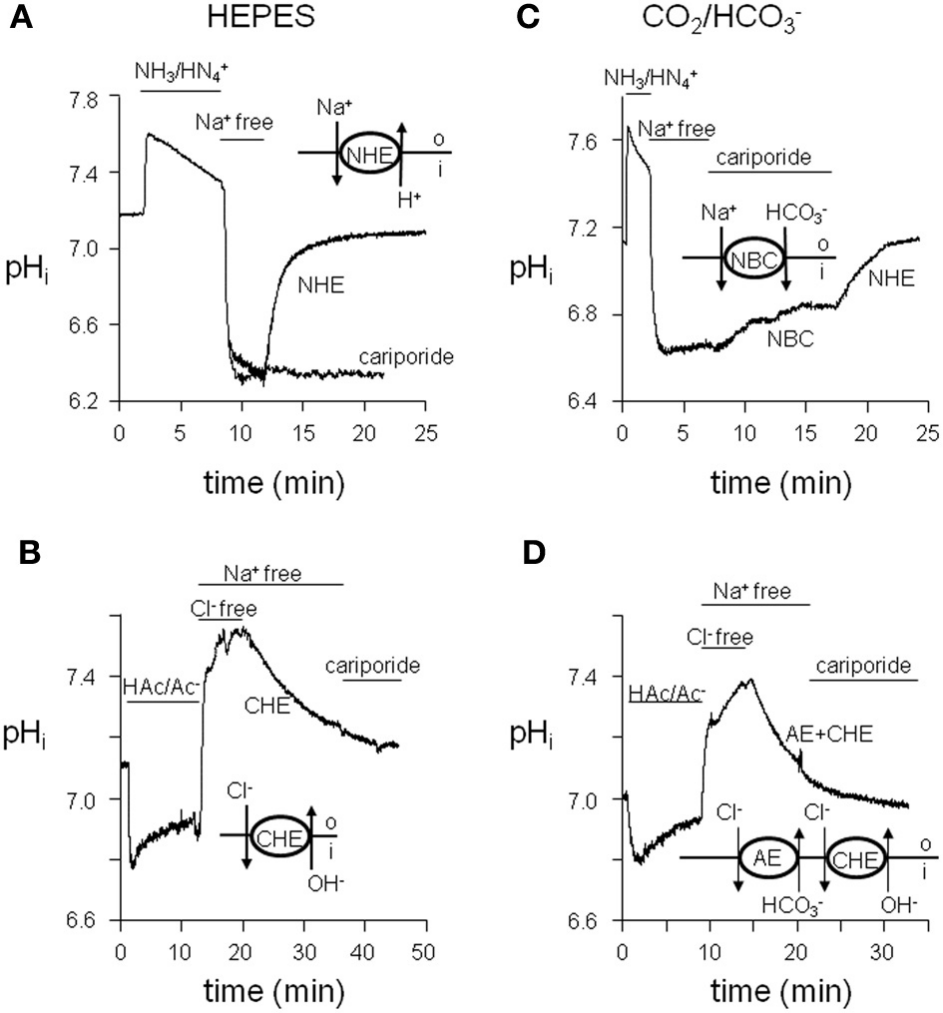
**Functional identification of sarcolemmal acid-base transporters in dog ventricular myocytes. (A)** pH_*i*_ recovery after an acute acid load, induced by withdrawal of 20 mM NH_3_/NH^+^_4_, under HEPES-buffered conditions proved extracellular Na^+^-dependent and cariporide sensitive. **(B)** pH_*i*_ recovery after an acute acid load, induced by withdrawal of 20 mM NH_3_/NH^+^_4_, under CO_2_/HCO^−^_3_-buffered conditions proved extracellular Na^+^-dependent and cariporide insensitive. **(C, D)** pH_*i*_ recovery from an acute alkaline load proved extracellular Cl^−^-dependent and extracellular Na^+^-independent and was induced by 40 mM HAc/Ac^−^ under HEPES-buffered conditions **(C)** and by 80 mM HAc/Ac^−^ under CO_2_/HCO^−^_3_-buffered conditions **(D)**.

Before studying the differences between normal and hypertrophied cAVB myocytes, we first identified what types of cardiac acid-base transporters are present. Acid-extruders and acid-loaders were activated by imposing acute acid and alkaline loads on the myocytes. Protocols are illustrated in Figure 1. In HEPES-buffered solutions, withdrawal of 20 mM NH_3_/NH^+^_4_ caused an acid load, the recovery from which was CO_2_/HCO^−^_3_-independent, but Na^+^-dependent and sensitive to 10 μM cariporide (Figure 1A). These characteristics are typical of NHE, presumably of the NHE-1 isoform. We cannot exclude a small contribution of the cariporide sensitive NHE-2 isoform, when present in dog cardiomyocytes. Withdrawal of 40 mM HAc/Ac^−^ caused an alkaline load, the recovery from which was CO_2_/HCO^−^_3_- and Na^+^-independent but Cl^−^-dependent (Figure 1B). These are hallmarks of the Cl^−^/OH^−^ exchanger (CHE).

In CO_2_/HCO^−^_3_-buffered solutions, a Na^+^-dependent and cariporide-insensitive acid load recovery was observed (Figure 1C). These characteristics are typical of the Na^+^-HCO^−^_3_ cotransporter (NBC). Recovery from an alkaline load upon withdrawal of 80 mM HAc/Ac^−^ was CO_2_/HCO^−^_3_-dependent and Cl^−^-dependent but Na^+^-independent (Figure 1D). These are the hallmarks of the Cl^−^/HCO^−^_3_ exchanger (anion exchanger, AE). The recovery from an alkaline load in CO_2_/HCO^−^_3_-buffered solutions is presumably mediated by both CHE and AE.

From these data we conclude that dog cardiac myocytes possess the four classical acid-base transporters NHE-1, CHE, NBC, and AE.

### Protocol used to study all acid-base transporters in one myocyte

**Figure 2:**
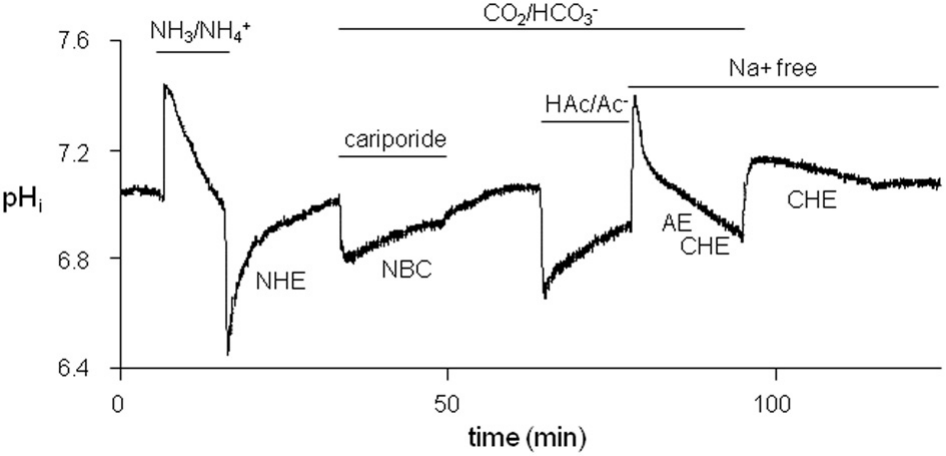
**Typical protocol used to successively examine the four sarcolemmal acid-base transporters in each myocyte**. Firstly, NHE-1 was studied after an acid-load induced by withdrawal of 20 mM NH^+^_4_/NH_3_. Secondly, NBC was studied, while NHE-1 was inhibited by cariporide, following a second weak acid load induced by replacement of a HEPES-buffered Tyrode's solution by a CO_2_/HCO^−^_3_-buffered Tyrode's solution. Thirdly, AE + CHE was studied in a Na^+^-free CO_2_/HCO^−^_3_-buffered saline solution after an alkaline load was induced by washout of 80 mM HAc/Ac^−^. Finally, CHE was studied in Na^+^-free conditions after an alkaline load was induced by replacing CO_2_/HCO^−^_3_-buffered with HEPES-buffered Tyrode's solutions.

To limit the time needed for studying all acid-base transporters, we designed a protocol for measuring NHE-1, NBC, AE, and CHE activity in one myocyte. A typical example is shown in Figure 2.

We first imposed an acid-load on a myocyte (NH_3_/NH^+^_4_ prepulse technique) under HEPES-buffered conditions. The acid-load was quickly alleviated by means of NHE-1 until pH_*i*, balanced_ was reached, the pH_*i*_ value at which an acid-base transporter is exactly balanced by the opposing *J*^+^_H_ flux (dpH_*i*_/dt equals 0). The myocyte was then subjected to a mild acid-load by replacing HEPES with 5% CO_2_/22.4 mM HCO^−^_3_ (pH 7.4) in the presence of 10 μM cariporide to inhibit NHE-1. The only acid-extruder active under these conditions is the HCO^−^_3_-dependent NBC. The NBC-mediated recovery was slow and incomplete, and stopped at the near neutral pH_*i*, balanced_ value. Washout of cariporide unblocked NHE-1, which led to further restoration of pH_*i*_ until the original steady-state pH_*i*_ value under CO_2_/HCO^−^_3_-buffered conditions once again was reached. Thereafter, the myocyte was alkaline loaded (HAc/Ac^−^ prepulse technique). The recovery from this load occurred quickly by means of AE and CHE. By choosing Na^+^-free condition at this stage of the experiment the Cl^−^-dependent acid-loaders were not opposed by the Na^+^-dependent acid extruders at neutral pH_*i*_ values. In this way their H^+^ equivalent flux at the steady-state pH_*i*_ could be determined. Finally, the myocyte was subjected to second and mild alkaline load (replacing CO_2_/HCO^−^_3_ with HEPES). From this alkaline load the myocytes recovered only very slowly by means of CHE and stopped recovering when CHE pH_*i*,balanced_ value was reached.

### Intrinsic buffering power (β_int_) in normal and hypertrophied cAVB myocytes

**Figure 3:**
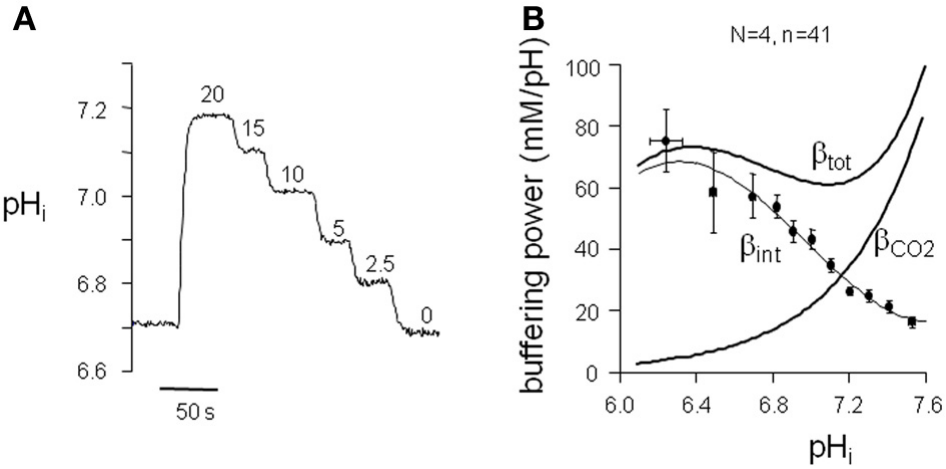
**pH_*i*_ dependence of buffering power in dog ventricular myocytes. (A)** Typical pH_*i*_ trace of a protocol to determine the intrinsic buffering power (β_int_) of ventricular myocytes. **(B)** The β_int_ values, within a 0.05 pH_*i*_ range, were averaged and plotted against the appropriate pH_*i*_ to construct the pH_*i*_-β_int_ relationship. The polynomial fit through the data is used to calculate *J*^+^_H_ under HEPES-buffered conditions. For *J*^+^_H_ under CO_2_/HCO^−^_3_-buffered conditions the calculated β_CO_2__ was added to β_int_ to arrive at β_tot_.

To compute cytoplasmic H^+^ equivalent fluxes (*J*^+^_H_) from pH_*i*_ recovery rates, we determined the pH_*i*_-dependence of the intrinsic buffering power (pH_*i*_-β_int_) in left and right ventricular myocytes of normal (left *N* = 2; *n* = 7 and right *N* = 2; *n* = 7) and cAVB (left *N* = 2; *n* = 15 and right *N* = 2; *n* = 12) dogs. Myocytes exposed to a series of NH^+^_4_/NH_3_ (20–2.5 mM) solutions showed a stepwise reduction in pH_*i*_ (Figure 3A). The pH_*i*_-β_int_ relationships did not significantly differ between myocytes isolated from the left and right ventricle, or between normal and cAVB hearts (data not shown). For this reason we pooled all data and calculated the average pH_*i*_-β_int_ relationship (*N* = 4; *n* = 41). The averaged pH_*i*_-β_int_ relationship was fitted with a polynomial equation (Figure 3B). Cytoplasmic *J*^+^_H_ was calculated at 0.05 pH_*i*_ intervals. Next, cytoplasmic *J*^+^_H_ were divided by the averaged surface-to-volume ratios and plotted as a function of the corresponding pH_*i*_ to construct sarcolemmal pH_*i*_-*J*^+^_H_ profiles of NHE-1 (pH_*i*_-*J*_NHE-1_), NBC (pH_*i*_-*J*_NBC_), AE + CHE (pH_*i*_-*J*_AE + CHE_), and CHE (pH_*i*_-*J*_CHE_).

### Sarcolemmal pH_*i*_-J^+^_H_ profiles in normal left and right ventricular myocytes

**Figure 4:**
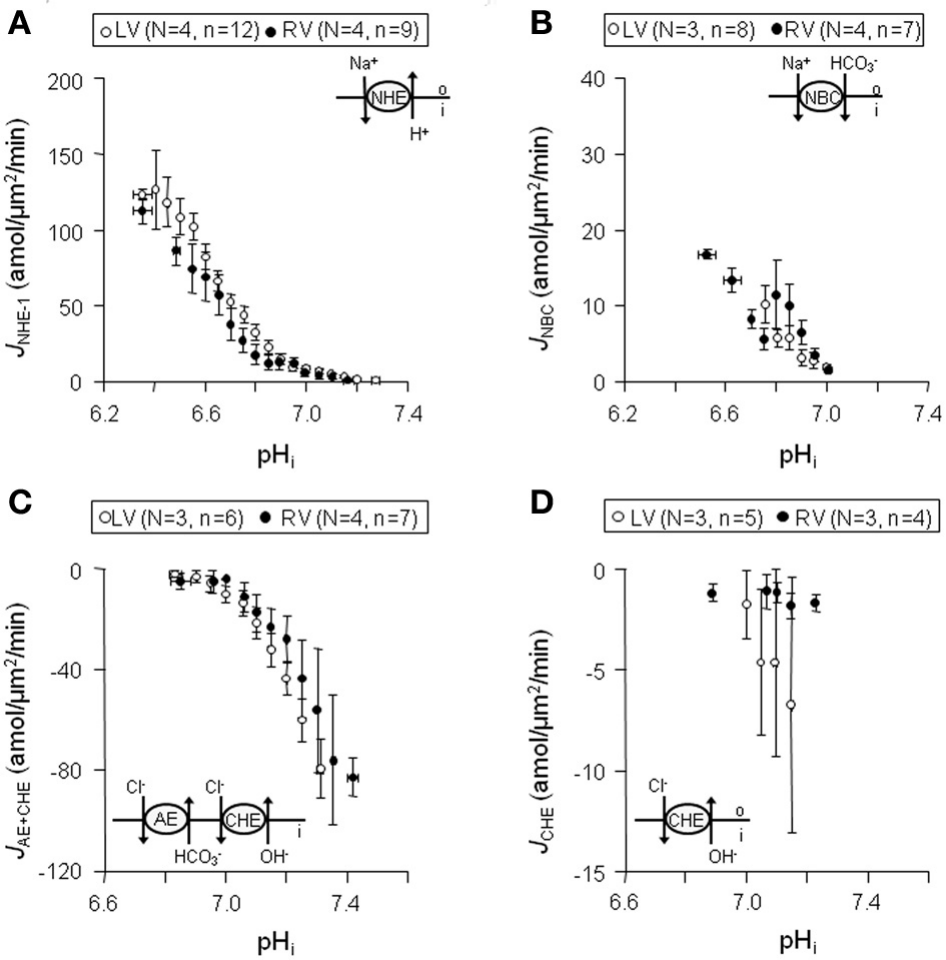
**Sarcolemmal *J*^+^_H_ through NHE-1, NBC, CHE + AE, and CHE in normal left and right ventricular myocytes**. Data obtained from left are shown in blank circles, whereas from right are indicated with filled circles. Panels **(A–D)** illustrates that sarcolemmal *J*_NHE-1_ **(A)**, *J*_NBC_ **(B)**,*J*_AE + CHE_ **(C)**, and *J*_CHE_ **(D)** are not significantly different throughout the whole pH_*i*_ range in left compared to right ventricular myocytes.

In both left (*n* = 12) and right (*n* = 9) ventricular myocytes from normal dog hearts (*N* = 4) sarcolemmal *J*_NHE-1_ (i) were equally large at acidic pH_*i*_ values, (ii) showed a comparable steep pH_*i*_-dependency, and (iii) had similar pH_*i*, balanced_ values (Figure 4A). Likewise, no differences in these three characteristics were found in sarcolemmal *J*_NBC_, between left (*N* = 3, *n* = 8) and right (*N* = 4, *n* = 7) ventricular myocytes (Figure 4B). Compared to NHE-1, (i) NBC fluxes were 77% smaller, (ii) NBC pH_*i*_-dependence was 7-fold less, and (iii) NBC pH_*i*, balanced_ value was ~0.2 pH more acidic. Thus, NHE-1 is the major acid extruder present in the dog myocardium.

The myocytes showed a rapid alkaline load recovery under CO_2_/HCO^−^_3_-buffered conditions. In left (*N* = 3, *n* = 6) and right (*N* = 4, *n* = 7) ventricular myocytes sarcolemmal *J*_AE + CHE_ was (i) equally large at alkaline pH_*i*_ values, (ii) showed a comparable pH_*i*_-dependency, and (iii) had a virtually identical pH_*i*, balanced_ value of around 6.8 (Figure 4C). Unlike sarcolemmal *J*_AE + CHE_, sarcolemmal *J*_CHE_ proved small and only weakly pH_*i*_ dependent. Again, no differences were observed between left (*N* = 3, *n* = 5) and right (*N* = 3, *n* = 4) ventricular myocytes (Figure 4D). Thus, AE is the major acid loader present in the dog myocardium and is largely responsible for alkaline load recoveries under CO_2_/HCO^−^_3_-buffered conditions.

We conclude that no differences exist in sarcolemmal pH_*i*_-*J*^+^_H_ profiles of NHE-1, NBC, AE, and CHE between myocytes from the left and right ventricle.

### Sarcolemmal pH_*i*_-J^+^_H_ profiles in normal and hypertrophied cAVB myocytes

**Figure 5:**
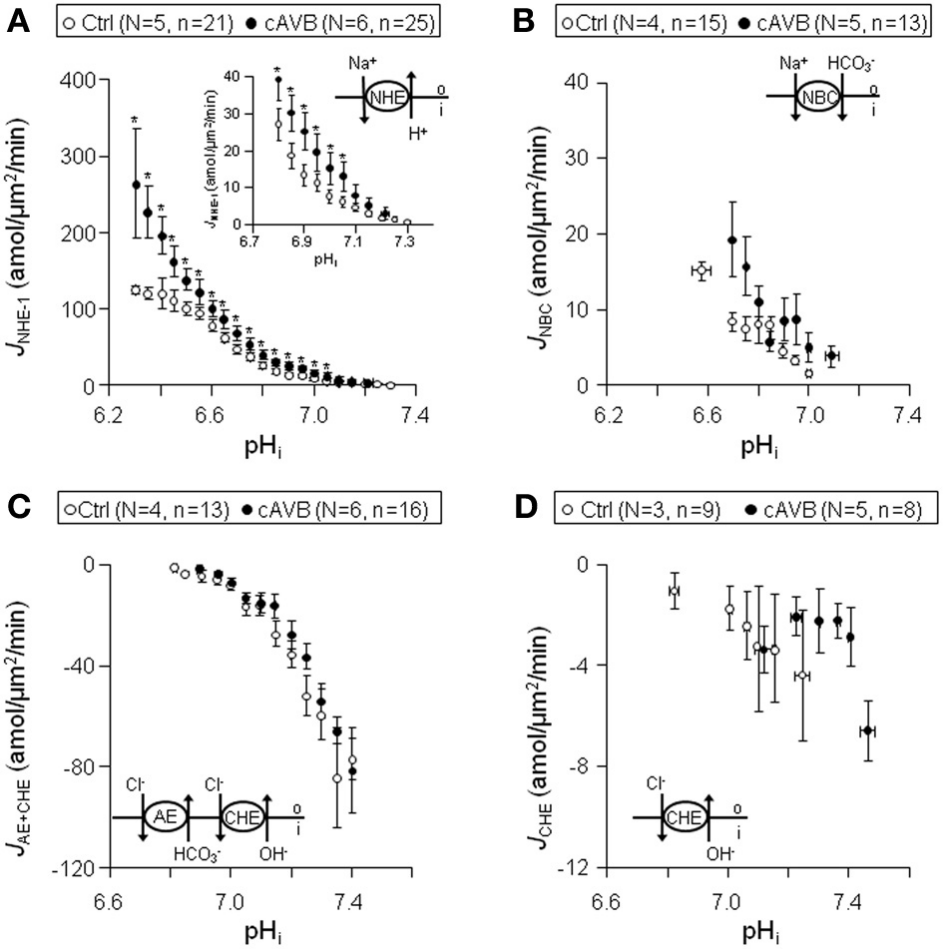
**Sarcolemmal *J*^+^_H_ through NHE-1, NBC, CHE + AE, and CHE in normal and hypertrophied cAVB ventricular myocytes**. Pooled data (LV + RV) obtained from normal (open circles) and cAVB myocytes (filled circles). Panels **(A–D)** illustrates sarcolemmal pH_*i*_-*J*_NHE-1_ **(A)**, pH_*i*_-*J*_NBC_ **(B)**, pH_*i*_-*J*_AE + CHE_ **(C)**, and pH_*i*_-*J*_CHE_ **(D)** profiles. Asterisks “^*^” indicate statistical significance.

Next, we examined whether the sarcolemmal pH_*i*_-*J*^+^_H_ profiles were changed in hypertrophied cAVB myocytes. Like in normal dog hearts, no differences were observed in the sarcolemmal pH_*i*_-*J*^+^_H_ profiles between left and right hypertrophied ventricular cAVB myocytes (data not shown). For this reason the pooled left and right sarcolemmal pH_*i*_-*J*^+^_H_ profiles of hypertrophied cAVB myocytes were compared to the pooled left and right sarcolemmal pH_*i*_-*J*^+^_H_ profiles of normal myocytes (Figure 5).

In hypertrophied cAVB myocytes the sarcolemmal *J*_NHE-1_ (Figure 5A), (i) was significantly increased at pH_*i*_ values more acidic than 7.05 (*P* < 0.05), the increase amounted to 65 ± 6.3% in the pH_*i*_ interval 6.3–7.2, (ii) showed a 75% steeper pH_*i*_-dependency, −4.4 ± 2.2 amol/μm^2^/min/pH unit (*n* = 25) vs. −2.2 ± 3.4 amol/μm^2^/min/pH unit (*n* = 21) (*P* < 0.05), and (iii) identical pH_*i*, balanced_ values of around 7.3. In contrast, no changes were observed in the sarcolemmal pH_*i*_-*J*_NBC_ (Figure 5B), pH_*i*_-*J*_AE + CHE_ (Figure 5C), and pH_*i*_-*J*_CHE_ (Figure 5D) profiles between normal myocytes and hypertrophied cAVB myocytes.

From these data we conclude that sarcolemmal *J*_NHE-1_ is increased in hypertrophied cAVB myocytes but otherwise there are no significant differences in sarcolemmal acid-base transport between normal hearts and compensated biventricular hypertrophied hearts.

### Sarcolemmal J^+^_H_ at resting pH_*i*_ in normal and hypertrophied cAVB myocytes

**Figure 6:**
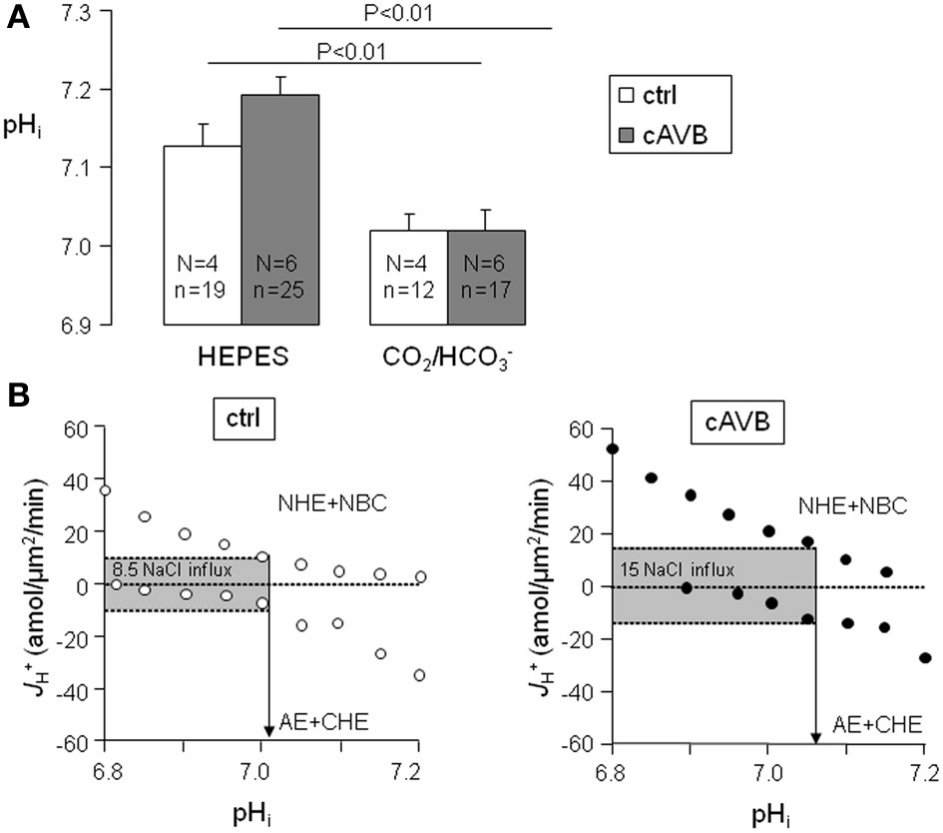
**Balanced cytoplasmic *J*^+^_H_ and sarcolemmal *J*^+^_H_ at steady-state pH_*i*_ in normal and cAVB myocytes. (A)** Steady-state pH_*i*_ values were obtained in normal (open bars) and cAVB (filled bars) myocytes either under HEPES or CO_2_/HCO^−^_3_-buffered conditions. Significant differences (*P* < 0.01), the number of animals (N) and observations (n) are indicated. **(B)** Total sarcolemmal *J*^+^_H_ of the acid extruders and the acid loaders are plotted against their pH_*i*_ in one graph to visualize their overlap at the steady-state pH_*i*_. *J*^+^_H_ of the acid extruders and acid loaders are balanced in normal myocytes at pH_*i*_ 7.01 (**B**, left panel) and in cAVB myocytes at pH_*i*_ 7.07 (**B**, right panel), causing respectively a sarcolemmal *J*^+^_H_ of 8.5 and 15 amol/μm^2^/min at these pH_*i*_ values.

When metabolic acid-base production is neglected, resting pH_*i*_ is defined by the balance of acid loading (*J*_CHE_ and/or *J*_AE_) and acid extrusion (*J*_NHE-1_ and/or *J*_NBC_). As is shown in Figure 6A, neither under HEPES-buffered conditions nor under CO_2_/HCO^−^_3_-buffered conditions the resting pH_*i*_ of normal and hypertrophied cAVB myocytes differed significantly. However, in both groups the resting pH_*i*_ was significantly more alkaline under HEPES-buffered conditions as compared to CO_2_/HCO^−^_3_-buffered conditions (*P* < 0.05). This difference indicates that in CO_2_/HCO^−^_3_-buffered solutions the acid loading action of AE drives pH_*i*_ more acidic. In the process NHE-1 and NBC increase their activity. At the new steady-state pH_*i*_, acid loading is balanced by acid extrusion, and the cell experiences increased NaCl influx as compared to HEPES-buffered conditions. We estimated the magnitude of the increased sarcolemmal NaCl influx from the sarcolemmal pH_*i*_-*J*^+^_H_ profiles of the four acid base transporters (Figure 4). In physiological, CO_2_/HCO^−^_3_-buffered solutions the sarcolemmal *J*^+^_H_, at the resting pH_*i*_ increased from 8.5 ± 1.5 amol/μm^2^/min in normal myocytes (Figure 6B, left panel) to 15 ± 2.4 amol/μm^2^/min in hypertrophied cAVB myocytes (Figure 6B, right panel). Thus, the sarcolemmal Na^+^ influx in CO_2_/HCO^−^_3_-buffered solutions at resting pH_*i*_ is increased by 76% in hypertrophied cAVB myocytes.

From these data we conclude that at resting pH_*i*_ there are substantial differences in NaCl loading between normal hearts and compensated biventricular hypertrophied hearts.

## Discussion

### Overview

We investigated acid-base transport in normal dog hearts, and in hearts from cAVB dogs with compensated hypertrophy. We first identified (Figures 1, 2) and characterized the sarcolemmal acid-base transporters. After determination of the intrinsic cytoplasmic buffering power (Figure 3) we constructed their pH_*i*_-*J*^+^_H_ profiles (Figure 4). NHE-1 and AE are the most potent acid extruder and acid loader, respectively, whereas the contribution of NBC and CHE to pH_*i*_ regulation is relatively small (Figure 4). Secondly, neither in normal myocytes nor in hypertrophied cAVB myocytes did we observed left and right differences in acid-base transport (Figure 4). Thirdly, we demonstrated that compensated biventricular hypertrophy is associated with increased sarcolemmal NHE-1 mediated H^+^ equivalent fluxes but unchanged NBC, AE, or CHE fluxes (Figures 5, 6). Consequently, at resting pH_*i*_ values, the amount sarcolemmal Na^+^ influx is significantly increased in hypertrophied cAVB myocytes (Figure 6).

### Acid-base transport of normal dog ventricular myocytes

In the past, the role of NHE-1 has been pharmacologically examined in dog hearts with respect to ischemia/reperfusion injury and ischemic preconditioning (Gumina et al., 2000, 2005; Oh et al., 2007), however nothing is known about its activity and pH_*i*_-sensitivity. In addition, molecular, pharmacological, and functional data on NBC, AE, and CHE in the dog myocardium are completely lacking. To gain insight in what type of acid-base transporters are present in dog ventricular myocytes we used the experimental approach previously published for guinea-pig and rabbit ventricular myocytes (Leem et al., 1999; van Borren et al., 2006). Except for minor quantitative differences there is good agreement between our data and those published for guinea pig and rabbit. In these species CHE contributes for 15–30% to the acid loading rate (Leem and Vaughan-Jones, 1998), whereas we found a contribution of less than 10% in dog ventricular myocytes. The remaining acid-loading capacity can be attributed to AE. It should be noted that in this study we cannot exclude a possible contribution of residual Cl^−^/HCO^−^_3_ exchange during the alkaline-load recovery under HEPES-buffered conditions.

In dog, NHE-1 is responsible for more than 80% to the total acid extrusion capacity. Similar transport rates were found in rabbit (65%) guinea-pig (Lagadic-Gossmann et al., 1992; Leem et al., 1999), rat (Le Prigent et al., 1997) and sheep purkinje fiber (Dart and Vaughan-Jones, 1992). The other 20% of the acid-extrusion capacity can be attributed to NBC. In this study we did not determine the relative contribution of the different NBC isoforms [electroneutral (NBCn) vs. electrogenic (NBCe)] (Yamamoto et al., 2005). Moreover, the expression levels and molecular identities of the acid-base transporters remain to be determined.

Differences in membrane protein expression levels between left and right ventricular myocardium have been described. For example the current densities of the repolarizing potassium currents, the transient outward potassium current (*I*_to_) and the delayed rectifier (*I*_Ks_), are larger in right ventricular myocytes (Volders et al., 1999; Di Diego et al., 2002) and may underlie the consequent shorter AP. Also left-right differences in Ca^2+^_*i*_ transients and contractions were observed. However, the smaller Ca^2+^_*i*_ transients and contractions of right ventricular myocytes were not related to differential expression of Ca^2+^_*i*_ handling proteins but to the shorter APs (Kondo et al., 2006).

In this study we demonstrated that steady-state pH_*i*_ as well as acid-base transport activity of NHE-1, NBC, AE, and CHE do not differ between left and right ventricular myocytes of dogs hearts.

### Decreased surface-to-volume ratio in hypertrophied cAVB myocytes

Comparison of membrane transport activities, e.g., acid-base transporters, between cells is only allowed when they exhibit an identical cell surface-to-volume ratio value. It has been shown that these values can differ between species and between developmental stages, but are similar between small and large myocytes from the same healthy hearts (Satoh et al., 1996). During cardiac hypertrophy myocyte shape alterations parallel changes in ventricular anatomy (Gerdes, 2002). Indeed, isolated ventricular myocytes of cAVB dogs with eccentric biventricular hypertrophy increased more in length (13–23%) than in width (4–13%). Irrespective whether cells were assumed cylindrical or brick shaped, the cell volume of hypertrophied cAVB myocytes (74–121%) increased more than the surface area (14–26%). Consequently, surface-to-volume ratio decreased in hypertrophied cAVB myocytes by 38–47%. In modest hypertrophy, cardiomyocytes are able to maintain a normal surface-to-volume ratio by increasing T-tubular surface area disproportionally (Gerdes, 2002). Apparently this is not the case in compensated hypertrophied hearts of dogs with cAVB. Changes in surface-to-volume ratios between normal and hypertrophied myocytes have also been found in rat with cardiac hypertrophy (Yamamoto et al., 2007).

### Acid-base transport in hypertrophied cAVB myocytes

Increased NHE-1 activity is observed in human hypertrophied cardiomyocytes (Yokoyama et al., 2000) and in hypertrophied myocytes from various animal models with hypertension or heart failure (Baartscheer, 2006). For instance in rats with monocrotaline-induced right ventricular failure (Chen et al., 2001), in diabetes type-2 rats (Darmellah et al., 2007) and spontaneous hypertensive rats (SHR) (Cingolani et al., 2003; Ennis et al., 2007) with heart failure, and in pressure and volume (Baartscheer et al., 2003; van Borren et al., 2006) and rapid pacing (Aker et al., 2004) induced rabbit models of heart failure NHE-1 activity is markedly enhanced. Both post-translational modulation and increased protein expression have been proposed to cause increased NHE-1 activity (Cingolani et al., 2008). In a number of these animal models chronic NHE-1 inhibition prevented (Kusumoto et al., 2001; Sandmann et al., 2001; Baartscheer et al., 2005) or even reversed the development of hypertrophy or heart failure(Camilion de Hurtado et al., 2002; Cingolani et al., 2003; Baartscheer et al., 2008; Baartscheer and van Borren, 2008). Moreover, transgenic mice with arterial hypertension (lacking the natriuretic peptide receptor type A) (Kilic et al., 2005) or heart failure (beta-adrenergic receptor over-expressing in the heart) (Engelhardt et al., 2002) developed cardiac hypertrophy and exhibit increased NHE-1 activity. Also in these transgenic animal models, NHE-1 inhibition prevented the development of cardiac hypertrophy and heart failure. Recently it became clear that increased cardiac NHE-1 activity alone is sufficient to activate hypertrophic Ca^2+^_*i*_ dependent signaling pathways (Nakamura et al., 2008) and to induce dilated hypertrophic cardiomyopathy (Coccaro et al., 2007; Nakamura et al., 2008). Together, these studies underscore the pivotal role of increased NHE-1 activity in the etiology of hypertrophy and heart failure.

In good agreement with to the aforementioned models of cardiac hypertrophy and heart failure, we demonstrated here that also in cAVB dogs with compensated biventricular hypertrophy, sarcolemmal NHE-1 flux is increased. It should be noted that our cAVB dogs are free from heart failure symptoms, hypertension and lack the substantial sustained or progressive increase in the levels of neurohumoral factors (Vos et al., 1998). This suggests that hypertrophy *per se* is associated with increased sarcolemmal NHE-1 activity. Mechanisms underlying increased sarcolemmal NHE-1 activity in compensated hypertrophied hearts require further investigation, but may include increased wall stretch.

AE, NBC, and CHE, have been studied less extensively than NHE-1. An increased cardiac AE exchange activity has been documented in SHR (Chiappe de Cingolani et al., 2001) and rabbits with heart failure (pressure and volume overload) (van Borren et al., 2006). In our model of compensated cardiac hypertrophy, AE proved unchanged. Perhaps hypertension or heart failure is essential for increased AE activity to occur. Contradicting data exist on NBC. In one study NBC activity proved increased in hypertrophied cardiomyocytes from pressure overloaded rats (Yamamoto et al., 2007), whereas in another NBC activity was unchanged in hypertrophied cardiomyocytes from rabbits with heart failure (pressure and volume overload) (van Borren et al., 2006). Here we add that compensated hypertrophy does not increase cardiac NBC activity. Moreover, like in rabbits with heart failure (van Borren et al., 2006) no significant changes in CHE activity was observed in hypertrophied cAVB myocytes.

### Cardiac acid-base transport can explain increased Na^+^ influx in hypertrophied cAVB myocytes

**Figure 7:**
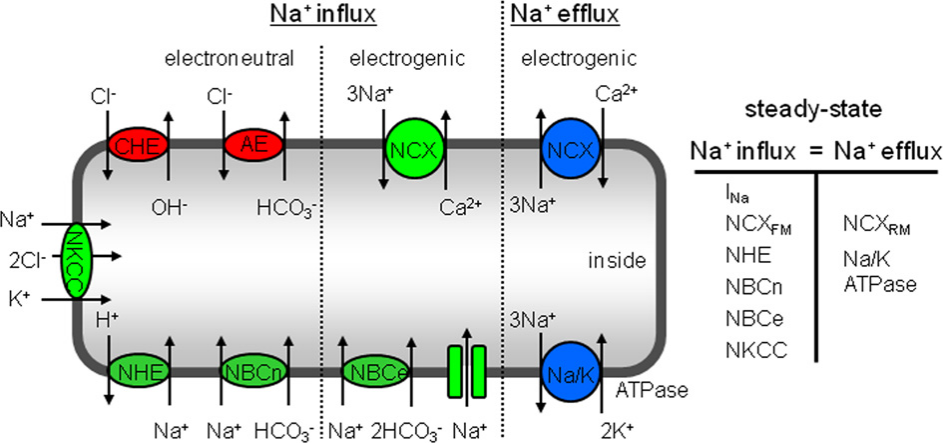
**Schematic overview of sarcolemmal transporters that regulate Na^+^_*i*_ concentration in cardiac myocytes**. At steady-state Na^+^ influx is exactly balanced by Na^+^ efflux and consequently Na^+^_*i*_ concentration remains unchanged. Na^+^ enters the cells through electroneutral transporters, such as the NKCC, NHE-1, and NBCn. The Na^+^-dependent acid extruders are stimulated by a continuous H^+^ supply via the Cl^−^-dependent acid loaders CHE and AE. Moreover, the electrogenic pathways through which Na^+^ enters is by NBCe, forward mode NCX and Na^+^ channels. The total Na^+^ influx is balanced by electrogenic Na^+^ extrusion mainly via the Na/K-pump and a minor part by reverse mode NCX.

The high Na^+^_*i*_ levels observed in hypertrophied cAVB myocytes have been attributed to a reduced affinity of the Na/K pump for Na^+^_*i*_ removal and to an increased Na^+^ influx (Verdonck et al., 2003). The latter was indirectly derived from Na/K pump currents, the major pathway for Na^+^ efflux that must equal Na^+^ influx under steady-state conditions in resting myocytes. A recent study revealed that hypertrophied cAVB myocytes exhibit both a reduced peak and late I_Na_, which excludes the sodium channels as a potential source for the enhanced Na^+^ influx (Antoons et al., 2008). Our data demonstrate that the balanced sarcolemmal *J*^+^_H_ flux (*J*_NHE-1 + NBC_ and *J*_CHE + AE_) at steady-state pH_*i*_ (Figure 6) is increased by 76%. The increased sarcolemmal Na^+^ influx through balanced acid-base transport needs to be fully compensated by the sarcolemmal Na/K pump. As previously reported by Verdonck et al., the maximal Na/K pump activity (pA/pF, a measure per μm^2^) remains unchanged and the affinity of the Na/K pump for Na^+^_*i*_ is reduced (Verdonck et al., 2003). Therefore, in hypertrophied cAVB myocytes the Na/K pump activity can only be sufficiently increased at higher Na^+^_*i*_ concentrations. The higher NHE-1-mediated Na^+^ influx calculated in this study (76%, Figure 6) closely matches the relative increase of pump current densities in hypertrophied cAVB myocytes (88%) observed by Verdonck et al. (2003). This implies that NHE-1 is the main pathway responsible for increased Na^+^ influx. Other influx pathways, such as NCX or Na-K-2Cl cotransporter (NKCC, Figure 7) may have a contribution as well. NCX, however, is an unlikely candidate because elevated Na^+^_*i*_ would rather promote the reverse mode and load the cell with Ca^2+^_*i*_, in particular in a setting of prolonged APs, as observed in cAVB (Pogwizd et al., 2003). Indeed, increased NCX-mediated Ca^2+^ loading was observed in hypertrophied cAVB myocytes (Sipido et al., 2000). NKCC may be an additional pathway for increased Na^+^ influx in cAVB, as documented previously for heart failure (Andersen et al., 2006). We cannot exclude a small contribution of NHE-2, when present in dog cardiomyocytes, since this transporter is also inhibited at the cariporide concentrations we used to identify NHE-1.

## Conclusion

Dogs exhibit four cardiac acid-base transporters, namely NHE-1, NBC, CHE, and AE. Their activities do not differ between left and right ventricular myocytes. Compensated hypertrophied hearts from cAVB dogs exhibit increased sarcolemmal *J*_NHE-1_ activity that almost fully explains the elevated Na^+^ influx in these hearts. Whether NHE-1 inhibition can prevent drug-induced Torsade-de-Pointes arrhythmias in cAVB dogs will be subject of future investigations.

### Conflict of interest statement

The authors declare that the research was conducted in the absence of any commercial or financial relationships that could be construed as a potential conflict of interest.
